# Micronuclei in germ cells of hybrid frogs from *Pelophylax esculentus* complex contain gradually eliminated chromosomes

**DOI:** 10.1038/s41598-020-64977-3

**Published:** 2020-05-26

**Authors:** D. Dedukh, S. Riumin, M. Chmielewska, B. Rozenblut-Kościsty, K. Kolenda, M. Kazmierczak, A. Dudzik, M. Ogielska, A. Krasikova

**Affiliations:** 1 Saint-Petersburg State University, Saint-Petersburg, Russia; 2 Amphibian Biology Group, Department of Evolutionary Biology and Conservation of Vertebrates, Faculty of Biological Sciences, University of Wrocław, Wrocław, Poland

**Keywords:** Chromosomes, Nuclear organization, Germline development, Cytogenetics, Polyploidy, Cell division, Genetic hybridization

## Abstract

In most organisms, cells typically maintain genome integrity, as radical genome reorganization leads to dramatic consequences. However, certain organisms, ranging from unicellular ciliates to vertebrates, are able to selectively eliminate specific parts of their genome during certain stages of development. Moreover, partial or complete elimination of one of the parental genomes occurs in interspecies hybrids reproducing asexually. Although several examples of this phenomenon are known, the molecular and cellular processes involved in selective elimination of genetic material remain largely undescribed for the majority of such organisms. Here, we elucidate the process of selective genome elimination in water frog hybrids from the *Pelophylax esculentus* complex reproducing through hybridogenesis. Specifically, in the gonads of diploid and triploid hybrids, but not those of the parental species, we revealed micronuclei in the cytoplasm of germ cells. In each micronucleus, only one centromere was detected with antibodies against kinetochore proteins, suggesting that each micronucleus comprises a single chromosome. Using 3D-FISH with species-specific centromeric probe, we determined the role of micronuclei in selective genome elimination. We found that in triploid LLR hybrids, micronuclei preferentially contain *P. ridibundus* chromosomes, while in diploid hybrids, micronuclei preferentially contain *P. lessonae* chromosomes. The number of centromere signals in the nuclei suggested that germ cells were aneuploid until they eliminate the whole chromosomal set of one of the parental species. Furthermore, in diploid hybrids, misaligned *P. lessonae* chromosomes were observed during the metaphase stage of germ cells division, suggesting their possible elimination due to the inability to attach to the spindle and segregate properly. Additionally, we described gonocytes with an increased number of *P. ridibundus* centromeres, indicating duplication of the genetic material. We conclude that selective genome elimination from germ cells of diploid and triploid hybrids occurs via the gradual elimination of individual chromosomes of one of the parental genomes, which are enclosed within micronuclei.

## Introduction

The maintenance of genome integrity is thought to be a crucial characteristic of eukaryotic organisms. However, some organisms have developed sophisticated mechanisms allowing them to selectively eliminate certain parts of their genome (sometimes up to 90%)^1–3^. The selective elimination of genetic material has been observed in a diverse range of animal groups, which includes chromatin diminution^1^, programmed DNA rearrangements^2,4–7^, the elimination of accessory or sex chromosomes^8,9^, and paternal genome elimination^10^. Additionally, selective elimination of genetic material in some interspecies hybrids seems to share similarities with the abovementioned processes, as genetic material destined for elimination is generally recognized, marked and selectively eliminated during specific stages of ontogenesis^3,11,12^. Interspecies hybrids exploit selective genome elimination to reproduce and are known to occur among animals that reproduce without recombination, collectively termed asexuals^12–14^. In such animals, gametogenesis is modified to produce gametes with an unreduced genome composition, and in the vast majority of these cases the genome of one parental species (usually paternal) is selectively excluded after fertilization (androgenesis, kleptogenesis, gynogenesis) or during gonocyte multiplication (hybridogenesis and triploid hybridogenesis)^11,13,15,16^. Nevertheless, the mechanisms underlying selective genome elimination are still a mystery.

Genome elimination that accompanies hybridogenetic reproduction is observed in interspecies hybrids of the European water frog (*Pelophylax esculentus*) complex^17,18^. Crossings of two parental species – the marsh frog *P. ridibundus* (RR) and the pool frog *P. lessonae* (LL) – produce the hybrid edible frog *P. esculentus*, in which adult individuals are either diploid (2n, RL) or triploid (3n, RRL and LLR)^19–22^. During gametogenesis, the genomes of diploid hybrid males and females undergo massive reorganization^18,23–25^. In particular, one parental genome (usually that of *P. lessonae*) is eliminated, while the remaining genome is duplicated to bypass normal meiosis, which leads to the production of haploid gametes^18,23,26–28^. Moreover, genome elimination has been observed in triploid hybrid frogs in a reproductive mode known as triploid hybridogenesis^13,29^. During triploid hybridogenesis, a single-copy genome is eliminated, and the two remaining genomes presumably undergo meiosis without duplication, leading to the formation of haploid gametes (i.e., in LLR hybrids, the R genome is usually eliminated; in RRL hybrids, the L genome is usually eliminated)^13,27,30^.

Reproduction via hybridogenesis (observed in *Poeciliopsis*^31^, *Hypseleotris*^32^*, Hexagrammos*^33^*, Bacillus*^34^, *Pelophylax*^17,26^) and triploid hybridogenesis (observed in *Misgurnus*^35^*, Squalius*^29,36^*, Cobitis*^37^*, Bufotes*^38^*, Pelophylax*^26,30^) has been found in hybrid organisms from various taxonomic groups. Nevertheless, the cytological mechanisms of parental genome elimination vary in each of these organisms, as elimination of the whole chromosomal set occurs either during gonocyte (germ cell) mitotic divisions^24,39,40^ or meiosis^41,42^. However, in studies of European water frogs, different groups have reported an absence of unipolar premeiotic genome elimination and observed aneuploid metaphase and occasional chromosomal lagging in dividing germ cells^23–25,43,44^. Moreover, in diploid and triploid hybrid frogs from various localities, the cytoplasm of the germ cells harbors micronuclei (previously known as nucleus-like bodies^24^) that contain DNA, probably from the eliminated genome^24,25,44,45^. A recent study showed that micronuclei are not associated with cell death but accumulate heterochromatin markers and degrade inside autophagosomes^25^. Moreover, Chmielewska *et al*.^25^ suggested the role of micronuclei in programmed DNA elimination through budding off the interphase nucleus. However, neither presence of chromosomes inside micronuclei nor their genomic identity (*P. ridibundus* or *P. lessonae*) has been proven so far for diploid and triploid hybrids. Taken together, mechanisms of selective genome elimination in water frog hybrids remain disputed. Here, we aimed to verify whether micronuclei act as vectors of selective genome elimination in diploid and triploid hybrids.

In current work we focused on diploid and triploid hybrid tadpoles obtained from artificial crosses of hybrid frogs with one another or the parental species. Using FISH with a species-specific centromeric probe, we identified the genomes in the micronuclei and gonocyte nuclei in gonads of diploid and triploid hybrid tadpoles. To confirm our FISH results, we detected kinetochores inside micronuclei in the cytoplasm of germ cells. Additionally, to describe possible mechanisms of genome elimination we analyzed gonocytes during metaphase. The obtained results allowed us to estimate the number and origin of the eliminated chromosomes in each micronucleus in the gonads of hybrid tadpoles.

## Results

We analysed the gonads of 106 diploid and triploid hybrid tadpoles obtained from 22 artificial crosses of diploid and triploid hybrids with each other or the parental species (Table S1, Table S2). The genome composition of the tadpoles was identified by FISH with centromeric or telomeric probes performed on metaphase plates from somatic tissues. Triploid tadpoles originated from crosses between diploid hybrid females and either triploid hybrid males with the LLR genotype or *P. lessonae* males (2 crosses). Diploid tadpoles were produced from various crossings of diploid and triploid hybrids as well as *P. lessonae* and *P. ridibundus* individuals, involving both females and males of all taxa: (1) both parents were hybrids, one 2n and one 3n (5 crosses); (2) one parent was a 2n (10 crosses) or 3n (2 crosses) hybrid, while the second parent came from a parental species; or (3) both parents came from the parental species (one crossing).

### Genome elimination in germ cells of diploid and triploid hybrid tadpoles occurs gradually

In the gonads of hybrid tadpoles and *P. ridibundus* individuals, gonocytes were identified with antibodies against the Vasa protein (Fig. S1a,b) and appeared as large cells with multiple nucleoli and less intensive chromatin staining compared to somatic cells. In all analysed hybrid tadpoles, we observed micronuclei (DAPI-positive chromatin bodies surrounded by the membrane) in the cytoplasm of germ cells. The frequency of germ cells with micronuclei ranged from 10 to 30% (Table S1). The number of micronuclei varied from 1 to 4 per individual germ cell.

**Figure 1:**
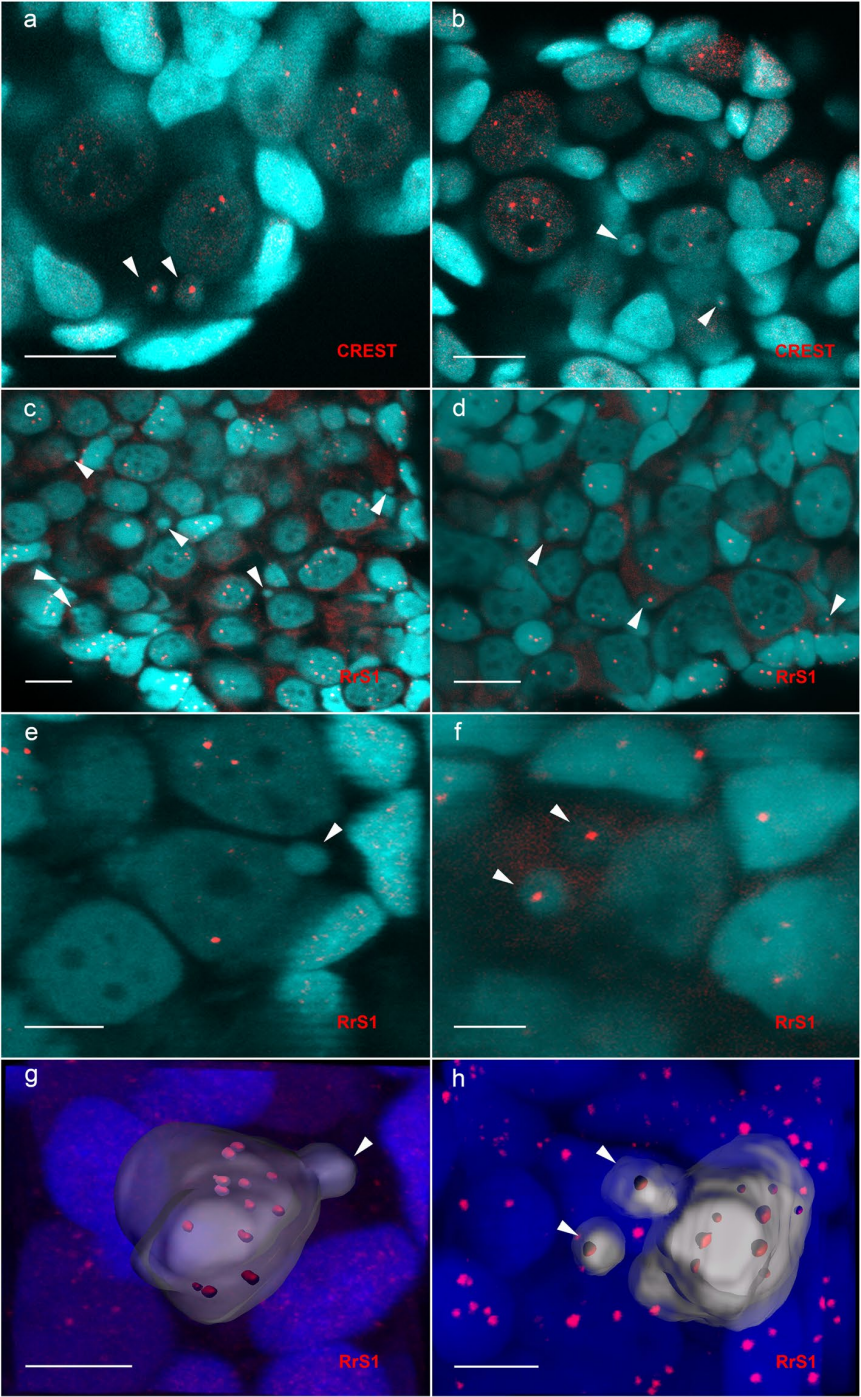
Micronuclei in germ cells (gonocytes) of tadpoles contain individual chromosomes selectively eliminated in diploid (**a,c,e,g**) and triploid (**b,d,f,h**) hybrids. **(a,b)** Whole-mount immunofluorescent staining with CREST serum in tadpole gonads revealed one kinetochore in each micronucleus. **(d,f)** 3D-FISH with a probe specific to the *P. ridibundus* RrS1 repeat revealed *P. ridibundus* centromeres in the cell nuclei in the gonads of diploid and triploid hybrids as well as in the micronuclei in the gonads of triploid hybrids. **(c,e)** *P. ridibundus* chromosomes were usually absent from the micronuclei in the gonads of diploid hybrids. Images (**a–f**) are single confocal sections of 0.8 µm in thickness. **(g,h)** 3D surface reconstruction of the germ cells and micronuclei depicted in (**e,f**). **(g)** 3D surface reconstruction clearly demonstrates the absence of signals in the micronuclei and 13 *P. ridibundus* centromeres (red) in the interphase nuclei (gray) of diploid hybrids. **(h)** 3D surface reconstruction shows the presence of 1 *P. ridibundus* centromere (red) in each micronucleus and 9 *P. ridibundus* centromeres (red) in the interphase nucleus (gray) of triploid LLR hybrids. Arrowheads indicate micronuclei. Scale bars for images (**a–d**) = 20 µm. Scale bars for images (**e–h**) = 10 µm.

Whole-mount immunostaining with anticentromere CREST serum against kinetochore proteins revealed centromere regions in the vast majority of micronuclei in all hybrid tadpoles studied (11 diploid and 2 triploid tadpoles) (Fig. 1a,b; Table S1). Furthermore, we usually observed one centromere in each micronucleus, and only 4% of micronuclei either lacked a centromere or exhibited two or three centromeres (Fig. 1a,b; Table S1). Thus, we concluded that each micronucleus usually comprises only one chromosome. This result indicates a gradual process of genome elimination, since the whole chromosome set of one of the parental species (n = 13 chromosomes) should be eliminated.

### In the germ cells of diploid hybrid tadpoles, micronuclei preferentially incorporate *P. lessonae* chromosomes

Here, a probe to the RrS1 centromeric repeat served as a species-specific marker allowing identification of the centromeres of *P. ridibundus* but not *P. lessonae* chromosomes by FISH. In previous studies, a probe to the RrS1 centromeric repeat marked 5 large chromosomes and 1 small chromosome^43,46,47^. According to our FISH protocol (see methods), we detected strong signals on 6–7 chromosomes, while the others exhibited weaker but detectable signals, suggesting differences in the accumulation of centromeric repeats between various *P. ridibundus* chromosomes (Fig. S2). The differences between our results and previous studies can be explained by either methodological modifications or a high level of heterochromatin polymorphism between different *P. ridibundus* populations.

**Figure 2:**
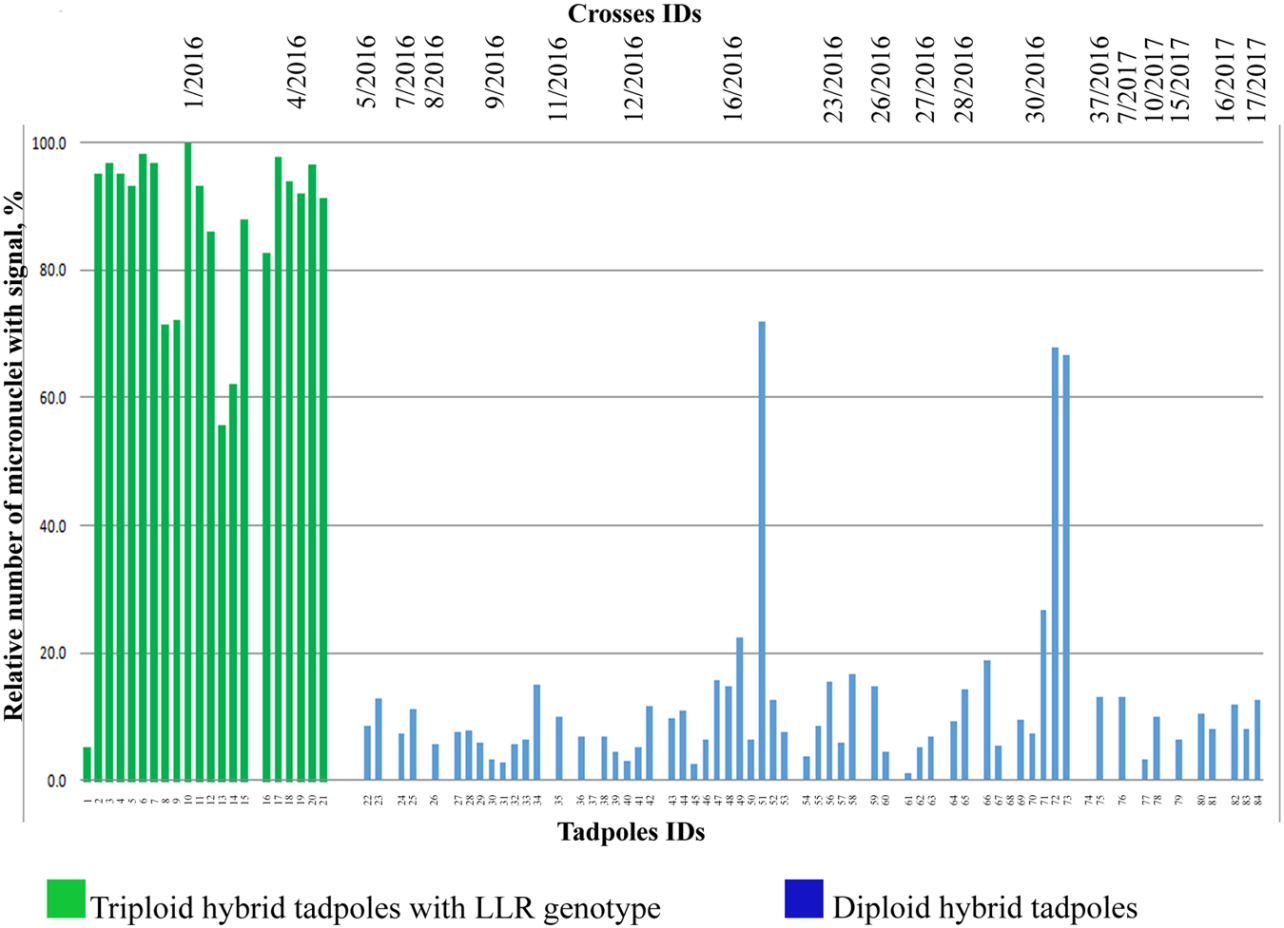
The relative number of micronuclei containing *P. ridibundus* chromosomes in diploid (marked blue) and triploid (marked green) hybrids obtained from various crosses.

FISH with an RrS1 centromeric probe specific to *P. ridibundus* centromeres was performed on the gonads of 63 diploid hybrids (Gosner^48^ stages 28–38) obtained from 18 different crosses (Table S1, Table S2). According to previous results and the current findings, centromere heterochromatin in the interphase nuclei of water frogs usually does not fuse (Fig. 1a,c,e,g)^46,47,49^. FISH with a centromeric probe or immunostaining with antibodies against kinetochore components revealed individual centromeres in interphase nuclei (Fig. 1a,c,e,g). Accordingly, in the majority of the interphase nuclei of gonocytes, we observed approximately 13 signals corresponding to *P. ridibundus* chromosomes (n = 13) (Fig. 1g). We examined 38,521 gonocytes and found 8,540 micronuclei in the gonads from 63 diploid tadpoles (Table S1). Among the micronuclei of 60 studied diploid hybrid tadpoles, we observed *P. ridibundus* centromeres in 694 micronuclei (approximately 9%), but did not reveal any signals in the remaining 7,449 micronuclei (91%) (Fig. 1c,e,g; Fig. 2; Table S1). Taking into account that each micronucleus usually comprises one centromere (and very likely one chromosome), we suggest that in diploid hybrid tadpoles *P. lessonae* chromosomes are preferentially eliminated via micronuclei. However, on average, 9% of the micronuclei from these tadpoles contained *P. ridibundus* chromosomes, indicating nonselective genome elimination in some populations of germ cells (Fig. 2). Accordingly, a small portion of the germ cell nuclei harboured fewer than 13 *P. ridibundus* centromeres. In the gonads of three tadpoles, we observed *P. ridibundus* chromosomes in approximately 70% of all analysed micronuclei, suggesting either an absence of selective genome elimination or the elimination of different genomes in various germ cell populations (Fig. 2; Table S1).

**Figure 3:**
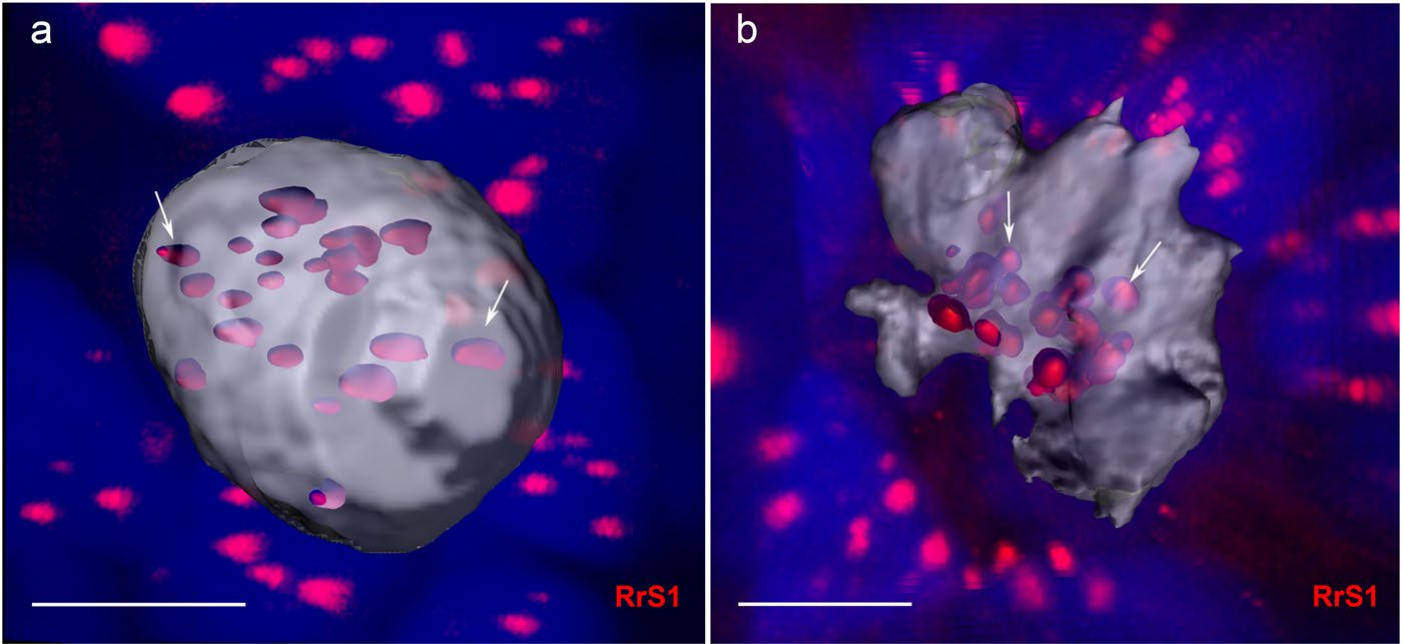
Germ cell interphase nucleus (**a**) and metaphase chromosome plate (**b**) with a doubled number of *P. ridibundus* chromosomes detected by FISH with a probe to centromeric RrS1 repeat. The 3D surface reconstruction indicates approximately 26 *P. ridibundus* centromeres in the gonocytes of diploid hybrid tadpoles (indicated by arrows). Scale bars = 10 µm.

Notably, in at least 10 diploid female hybrid tadpoles in developmental stages 33–38 (according to Gosner^48^) and gonadal development stages 4–5 according to Ogielska and Kotusz^50^, we detected individual cells (approximately 4–8 per individual) with 26 *P. ridibundus* centromeres (Fig. 3a,b). We suggest that such gonocytes with a doubled number of *P. ridibundus* chromosomes appeared as a result of genome duplication.

### In the germ cells of triploid hybrid tadpoles, micronuclei preferentially incorporate *P. ridibundus* chromosomes

FISH with an RrS1 centromeric probe specific to *P. ridibundus* centromeres was performed on the gonads of 21 triploid hybrid tadpoles with LLR genotype obtained from 2 different crosses (Table S1, Table S2). We found that the number of micronuclei containing *P. ridibundus* chromosomes was significantly higher in triploid hybrids as compared to diploid hybrid tadpoles (p < 0.01). We investigated 9,546 germ cells from the gonads of all triploid hybrid tadpoles with an LLR genotype and detected 2,375 micronuclei (Table S1). Detailed analysis showed that the gonads of 18 triploid hybrid tadpoles with LLR genotype contained 1,898 micronuclei (92%) with *P. ridibundus* chromosomes (Fig. 1d,f,h; Fig. 2; Table S1). Approximately 8% of micronuclei did not contain *P. ridibundus* chromosomes (Fig. 1d,f,h; Fig. 2; Table S1). Moreover, in germ cell nuclei, the number of *P. ridibundus* chromosomes was almost always lower than 13 and usually ranged from 4 to 11 (Fig. 1h). In two tadpoles, we detected *P. ridibundus* chromosomes in approximately 59% of all observed micronuclei, suggesting either an absence of selective genome elimination or the elimination of different genomes in different germ cells (Fig. 2; Table S1). Only one triploid tadpole with LLR genotype contained small number of *P. ridibundus* chromosomes, which were found in 5% of all observed micronuclei, suggesting the preferential elimination of *P. lessonae* chromosomes (Fig. 2; Table S1).

### Misaligned chromosomes can be selectively eliminated during metaphase

**Figure 4:**
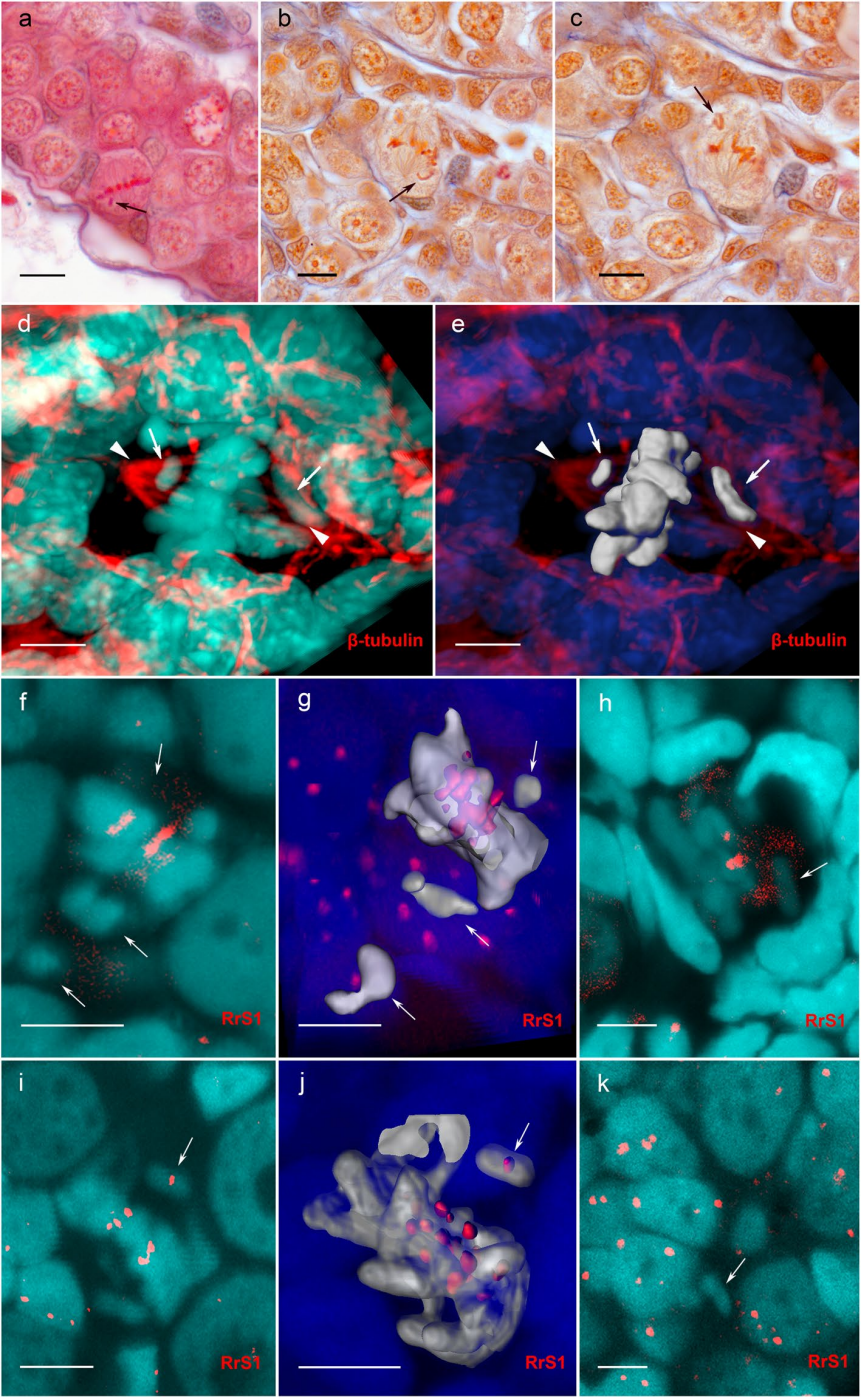
Metaphases with misaligned chromosomes in the gonads of diploid hybrids. **(a–c)** Histological sections of gonads show metaphase plates with misaligned chromosomes. **(d,e)** 3D surface reconstruction of the metaphase plate after immunofluorescent staining with antibodies against b-tubulin performed in frozen tissue sections from the gonads of hybrid females. Misaligned chromosomes (indicated by arrows) unable to attach to the spindle (indicated by arrowheads). **(f–j)** Misaligned *P. lessonae* chromosomes **(f,g,h)** and *P. ridibundus* chromosomes **(i,j)** during the metaphase stage identified by FISH with a probe to centromeric RrS1 repeat. Images (**f,h,i**,**k**) are single confocal sections of 0.8 µm in thickness. **(g,j)** 3D surface reconstructions of metaphase chromosomes depicted in panels (**f,i**), which specifically show misaligned *P. lessonae* **(g)** and *P. ridibundus* **(j)** chromosomes. **(k)** Individual *P. lessonae* chromosomes located in the cytoplasm of the cells during interphase. Scale bars for images A-C = 20 µm. Scale bars for images D-K = 10 µm.

Although numerous micronuclei were present within the gonads of hybrid tadpoles and were observed in approximately 30% of germ cells, we found 19 cases of misaligned chromosomes during the mitotic division of germ cells in diploid hybrids (Fig. 4a–j; Table S1). Misaligned chromosomes exhibited an abnormal or no connection to the spindle (Fig. 4a–e). Notably, the misaligned chromosomes corresponded to *P. lessonae* chromosomes in 13 cases, whereas they corresponded to *P. ridibundus* chromosomes in only two cases (Fig. 4f–j). Moreover, in germ cells of two tadpoles, we observed individual *P. lessonae* chromosomes located in the cytoplasm and separated from the interphase nucleus (Fig. 4k).

## Discussion

Here, we clearly demonstrated that in hybrid European water frogs chromosomes are gradually eliminated from the gonocyte nuclei via micronucleus formation. Each micronucleus contains only one chromosome, while the majority of gonocytes are aneuploid until they eliminate all chromosomes of one of the parental genomes. We showed that in the majority of cases, the elimination of chromosomes is selective; i.e., the chromosomes of one of the parental species are eliminated. Several cases of misaligned chromosomes during gonocyte mitosis were observed. Genome identity of misaligned chromosomes coincided with the chromosome composition of micronuclei. In addition, genome duplication in the germ cells of hybrid tadpoles was revealed.

### Genome duplication in germ cells of diploid hybrids

In addition to genome elimination, we observed germ cells with an increased number of *P. ridibundus* chromosomes in diploid hybrids (Fig. 3a), suggesting the doubling of *P. ridibundus* genome, similar to observations made by Heppich and Tunner^18^. Such cells were detected in tadpoles at stages 33–38 according to Gosner^48^, which corresponds to stages 4–5 of gonadal development according to Ogielska and Kotusz^50^. In accordance with suggestions that in diploid hybrids premeiotic genome doubling occurs after the elimination of the *P. lessonae* chromosomes^23,26^, we detected 26 *P. ridibundus* but no *P. lessonae* chromosomes in certain gonocyte metaphases (Fig. 3b). However, proper cytogenetic markers are still unavailable for the *P. lessonae* genome; thus, we cannot check whether some *P. lessonae* chromosomes are still present in the gonocyte nuclei during interphase.

According to the classical scheme of hybridogenesis in water frogs, genome endoreplication is usually absent in triploids^27^. In triploid hybrid tadpoles, the number of *P. ridibundus* chromosomes usually ranged from 4 to 11 suggesting the absence of cells with duplicated genomes. However, oocytes with a duplicated genome were occasionally found among triploid hybrids from R-E populations^51^. Thus, the frequency of genome duplication in the gonocytes of triploid hybrids requires further investigation.

Premeiotic genome doubling has been suggested to be an almost universal mechanism of unreduced egg formation in the asexual hybrids of vertebrates, invertebrates and some plants^11,12,52^. Indeed, in the majority of cases, genome replication has been inferred after the identification of the genome composition in gametes^11–14^. In contrast, an alternative mechanism has been described for *Carassius* hybrids: germ cells have been shown to fuse with each other during gametogenesis, leading to the formation of gametes with doubled chromosome set^53^. However, in diploid and triploid *P. esculentus* tadpoles, neither we nor other researchers^24,25,44,45^ have observed the fusion of germ cells. Thus, we propose that the most likely mechanism of the formation of germ cells with a doubled number of *P. ridibundus* chromosomes is either genome endoreplication (cell cycle without mitosis) or endomitosis (mitosis without chromosome segregation).

### Nucleus budding and chromosome lagging as mechanisms of genome elimination in *P. esculentus*

In the present study, we demonstrated for the first time the correspondence between the genome origin of misaligned chromosomes and the genome composition of micronuclei in the same gonocytes of diploid hybrids. Together with our recent results showing that micronuclei can form via budding from interphase nuclei^25^, our current finding that misaligned *P. lessonae* chromosomes appear during the mitosis of gonocytes of diploid hybrids confirms their loss during anaphase (Fig. 4). Moreover, in studied hybrids, misaligned chromosomes show inability to attach to the spindle and are located outside the metaphase plate during mitosis (Fig. 4). Such chromosomes often tend to lag during anaphase and are not incorporated into the nuclei after mitotic division, causing aneuploidy in different cell cultures, moreover, they are observed at a high frequency in the presence of stress factors^54,55^. Furthermore, misaligned chromosomes in plant hybrids are often incorporated in micronuclei during selective genome elimination^56–59^. Thus, in accordance with other studies^23–25^, here we suggest that in water frog hybrids, the genome can be eliminated not only via the budding off the interphase nucleus but also through the inability of individual chromosomes to attach to the spindle, causing them to lag during the mitotic divisions of gonocytes.

The earlier suggestion that the chromosomes of *P. lessonae* can lag during the mitotic division of gonocytes was rooted to the fact that chromosomes of parental species exhibit different number of copies of the centromeric repeat^44^. Furthermore, the divergence of species-specific centromere-binding proteins frequently leads to the inability of chromosomes of one parental species to attach to the spindle, causing them to lag and eliminate in interspecies hybrids^57–60^. Selective chromosomal loss was also observed in artificial and natural plant hybrids between species with different variants of centromeric histone CENP-A^57,61,62^. Moreover, the elimination of such chromosomes can be gradual via aberrant loading of centromeric proteins from one parental species to the centromeres of the another^61^. Failure to attach to the spindle caused by the aberrant uploading of centromeric histones has also been documented for the fragments of holocentric chromosomes that are lost during chromatin diminution in somatic cells of *Parascaris univalens*^63^. Another aberration affecting centromere stability and function is known to be related to centromere-associated noncoding RNAs^60,64,65^. In addition to misaligned chromosomes, we also detected individual chromosomes in the cytoplasm of certain germ cells while the main nucleus was in interphase, which could indicate an inability of such chromosomes to incorporate to the main nucleus after mitosis. Although the underlying mechanism of misaligned chromosome formation in water frog hybrids requires further investigation, it seems that there are many complex avenues causing selective genome elimination.

### Micronuclei include single chromosomes that are gradually eliminated from the nucleus

**Figure 5:**
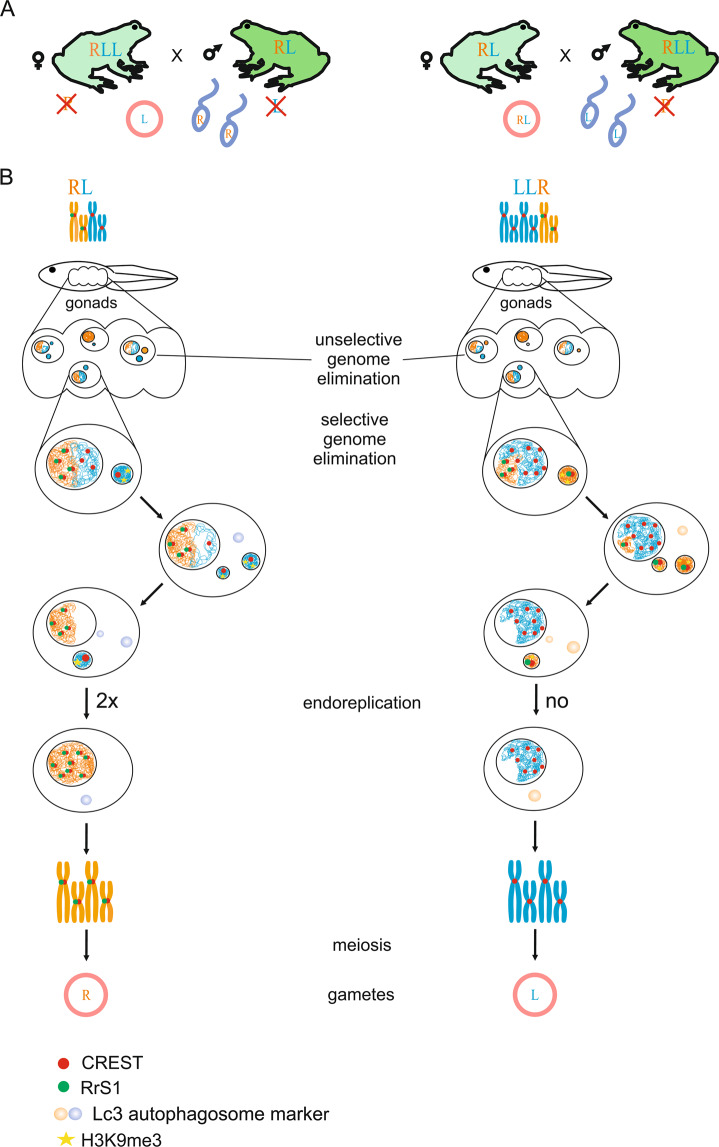
Schematic overview of selective genome elimination in diploid (left) and triploid (right) hybrid tadpoles. **(A)** Classical scheme of the reproduction of diploid and triploid hybrid frogs. **(B)** Suggested simplified scheme of gradual chromosome elimination via micronucleus formation in diploid and triploid hybrids. Individual chromosomes were detected via the visualization of centromeric regions with CREST antibodies (red circle) and FISH with a probe specific to the *P. ridibundus* RrS1 centromeric repeat (green circle). Each micronucleus usually comprises only one chromosome. In the micronuclei, chromatin accumulates heterochromatin markers, indicated by the yellow asterisk (according to Chmielewska *et al*.^25^). Subsequently, micronuclei accumulate autophagosome marker LC3 (transparent blue micronuclei) and undergo degradation (according to Chmielewska *et al*.^25^). In diploid hybrid frogs, after the elimination of the *P. lessonae* genome, *P. ridibundus* chromosomes undergo endoreplication, leading to a doubled number of chromosomes. Cells in which both genomes are eliminated probably die.

By the identification of the chromosomes contained within micronuclei and interphase nuclei of the same gonocytes, we confirmed the role of micronuclei in selective genome elimination during early gametogenesis in hybridogenetic diploid and triploid water frog hybrids for the first time (Fig. 5). Using FISH with the species-specific probe to the centromeric region and immunofluorescent detection of kinetochore proteins we showed that micronuclei usually contained only one chromosome. Regardless of the cross type, all diploid hybrid tadpoles preferentially eliminated *P. lessonae* chromosomes, while triploid LLR hybrids preferentially eliminated *P. ridibundus* chromosomes (Fig. 5; Table S1, Table S2). In diploid hybrids, we also observed misalignment of *P. lessonae* chromosomes that corresponds to the genome composition of the majority of micronuclei. Moreover, the genome composition of interphase nuclei was in agreement with the genome composition of micronuclei. In triploid LLR hybrids, we observed germ cells with a decreased number of *P. ridibundus* chromosomes usually ranged from 4 to 13 chromosomes, while in diploid hybrids, we observed germ cells with all *P. ridibundus* chromosomes. Our results suggest that during genome elimination gonocytes are aneuploid until one of the parental genomes will be completely eliminated from the nuclei. As we usually observe from 1 to 3 micronuclei (this study and^24,25,44,45^) and/or misaligned chromosomes as well as decreased number of *P. ridibundus* chromosomes in gonocytes of triploid LLR hybrids, we assume that several (up to 13) gonocyte divisions are required to eliminate one of the parental genomes. However, chromosome elimination through budding of the interphase nucleus^24,25^ can speed up this process. Continuous chromosome elimination through several mitotic divisions was detected in somatic cells of plant hybrids^56–59^, during elimination of supernumerary chromosomes^66^ and through programmed DNA rearrangement^1,2,7,67^.

Both chromosomal budding and lagging have been frequently observed in somatic cells of plant hybrids during early development^58,59^. In contrast to hybrid plants, genome elimination in water frog hybrids occurs only in germ cells during the specific stage of their gonadal development while genomes of somatic cells remain stable. In hybrid plants, Gernand *et al*.^58^ observed lagging chromosomes and chromosomal fragments without centromeres, while centric chromosomal fragments were usually extruded via budding from the interphase nuclei. Such chromosomes can be rearranged within micronuclei to form new chromosomes which can be inherited^68^. However, our findings suggest that in water frog hybrids, the vast majority of micronuclei contain centromeres, indicating the encapsulation of the entire chromosomes but not their fragments in micronuclei. This observation, together with chromatin condensation in the micronuclei, could explain the observed variation in the size of micronuclei (this study^25,44,45^).

In contrast to plant hybrids, gradual chromosome elimination occurs rarely among hybrid vertebrates and is well described only for water frog hybrids to date. In the majority of known asexual hybrids and cases of paternal genome elimination, whole sets of parental chromosomes are eliminated via a single mitotic division immediately after insemination (ambystomes^69^, molluscs from the *Corbicula* genus^70^, hybrid *Carassius* species *Carassius gibelio*^71^), during the mitotic division of gonial cells (hybridogenetic *Poeciliopsis*^39^) or during meiosis (in the loach *Misgurnus anguillicaudatus*^42^ and probably *Squalius alburnoides*^41^). In the other asexuals (*Bufotes*^38^ and stick insects from the *Bacillus* genus^40^), the genome elimination occurs before meiosis but its cellular mechanisms remain undiscovered.

The results for the majority of diploid and triploid hybrid tadpoles were highly concordant with the genome predicted to be eliminated according to the accepted model of hybridogenesis^26,27^ in adult hybrid frogs. Nevertheless, in both diploid and triploid LLR hybrids, we detected a small portion (around 9%) of micronuclei including different chromosomes than expected from the classical scheme of hybridogenesis. Presence of such micronuclei indicates irregularities in genome elimination in some gonocytes and may lead to the previously detected increased level of gonial cell death in the gonads of hybrids^72^. On the other hand, the potential ability of such gonocytes to proceed beyond meiosis can lead to a simultaneous production of gametes with various genome composition by individual hybrid frogs frequently detected in various populations^20,26,30,43,44,73,74^.

We stress that the following findings imply complicated mechanisms involved in genome elimination in water frog hybrids (Fig. 5): (1) chromatin heterochromatinization occurs within micronuclei^25^; (2) kinetochores are still present within micronuclei; and (3) *P. ridibundus* chromosomes are preferentially eliminated in triploid LLR hybrids, while *P. lessonae* chromosomes are usually eliminated in diploid RL and triploid RRL hybrids^20,26,44,45,51,74^.

### Recognition of genetic material is crucial for its selective elimination

The molecular mechanisms of selective genome elimination remain mostly unknown. Similar to the majority of the known cases of the selective elimination of genetic material, we suggest that in water frog hybrids one of the parental genomes should be recognized, marked, eliminated from the nucleus, and finally degraded. According to recent studies and the current work, selective genome elimination in *P. esculentus* hybrids involves chromosomal budding from the interphase nucleus and lagging during gonocyte divisions, leading to the formation of micronuclei, which are likely degraded via autophagy^24,25,44,45^. However, key components of genome elimination are not known for hybrid animals as well as almost all other cases in which the selective elimination of genetic material has been observed. The crucial role of noncoding RNA in recognizing DNA sequences designed for removal (in Oligohymenophorea^4^) or retention (in Spirotrichea^5^) has been previously demonstrated for only two classes of ciliate protozoa. To explain the mechanism of selective genome elimination, we previously hypothesized that in hybrid germ cells, retrotransposons can become activated in the genome, which is not protected by maternally inherited short noncoding RNAs from the oocyte^51^. However, in the current study, we have shown that regardless of the parent-of-origin effect, diploid hybrid tadpoles usually eliminate the L genome, while triploid LLR hybrid tadpoles usually eliminate the R genome. Based on studies of selective paternal genome elimination in *Coccilla* insects^9,75^, we propose the possibility of competition between the *P. ridibundus* and *P. lessonae* genomes that induces elimination and the development of anti-elimination mechanisms, but only in germ line cells. We can hypothesize that noncoding RNA from one genome can distinguish DNA sequences from the other genome as alien and prevent them from functioning, leading to their removal. Alternatively, the RNA of one genome can protect its genome from elimination and lead to the elimination of the unprotected genome.

## Materials and Methods

### Samples studied

European water frogs were collected from drainage areas in northwestern and southwestern Poland. *P. ridibundus* (N = 4) and *P. lessonae* (N = 10) individuals were captured from R-E (Ruda Milicka) and L-E systems (Sanie, surroundings of Wroclaw and Urwitalt in Mazury), respectively. Diploid hybrids (N = 13) and triploid hybrids with an LLR genome composition (N = 7) were collected from two separate E-E systems (Wysoka Kamieńska and Uciechów). The parental species and all hybrids except for triploid frogs with the LLR genotype were represented by both sexes. All manipulations with animals were carried out in accordance with national and international guidelines.

### Crossing experiments

Artificial crosses were carried out according to the protocol described in Berger *et al*.^76^ after hormonal stimulation. Females were injected intraperitoneally with salmon luteinizing hormone-releasing hormone (LHRH, H-7525.0001, Bachem) in amphibian PBS (APBS, pH 7.4, 11.2 mM NaCl, 0.22 mM KCl, 0.8 mM Na_2_HPO_4_, 0.14 mM KH_2_PO_4_) at a dose of 6.25 mg/kg of body weight. After the females had spawned, their eggs were fertilized with a homogenate of dissected testes from male frogs. Males and females were euthanized after being anaesthetized in a 0.5% solution of ethyl 3-aminobenzoate methane sulfonate (MS-222, Sigma Chemical Co.) in APBS, followed by tissue collection for chromosomal analysis. From stage 28 according to Gosner^48^ (hind limb bud development) to stage 42 (forelimb emergence), 5–20 tadpoles were randomly collected from each clutch each week. The tadpoles were placed in an anaesthetic solution (0.15% MS 222) preceding the dissection of their gonads, gills and intestines. Gonadal tissues were fixed in 2% paraformaldehyde in 1× PBS for 90 min at room temperature (RT) and then kept in 1× PBS with 0.02% NaN_3_ until use.

We analysed 106 diploid and triploid hybrid tadpoles obtained from 22 artificial crosses of diploid and triploid hybrids with each other and parental species (Supplementary Table 1). The tadpole genome composition was identified by FISH with centromeric or telomeric probes performed on metaphase plates from somatic tissues according to^44,49^.

### Probe labelling

The labelling of the RrS1 probe specific to the pericentromere tandem repeat of *P. ridibundus*^46^, not *P. lessonae*, was carried out by PCR (annealing temperature 62 °C) with the genomic DNA of *P. ridibundus* and the following primers according to a previously published protocol^49^:

Forward: 5′-AAGCCGATTTTAGACAAGATTGC-3′

Reverse: 5′-GGCCTTTGGTTACCAAATGC-3′

The probes were labelled with biotin-16-dUTP (Roche). In addition, a Cy3-labelled oligonucleotide probe (5′- AAGCCGATTTTAGACAAGATTGC-3′) specific to the *P. ridibundus* RrS1 pericentromeric sequence was used. A single-stranded oligonucleotide telomeric probe (TTAGGG)_5_ conjugated with biotin or fluorochrome Cy3 was also used to identify species.

### Fluorescent *in situ* hybridization

Fluorescent *in situ* hybridization was performed according to previously published protocols^44,45,49^. For hybridization, we used either a labelled probe generated from genomic DNA by PCR or a directly labelled oligonucleotide probe for the RrS1 repeat; however, the best results were provided by a PCR-labelled probe. The hybridization mixture contained 50% formamide, 10% dextran sulphate, 2× ЅЅС, 5 ng/μl labelled probe and a 10–50-fold excess of tRNA. In the case of the oligonucleotide probe, the hybridization mixture contained 40% formamide, 10% dextran sulphate, 2× ЅЅС, 5 ng/μl labelled probe and a 10–50-fold excess of tRNA. Joint denaturation of the probe and chromosomal DNA on the slides was performed at 75 °C for five minutes. The slides were incubated for 12–24 hours at room temperature (RT). After hybridization, the slides were washed in 0.2× SSC at 50 °C. Oligonucleotide-probed slides were washed in 2× ЅЅС at 42 °C. Biotin was detected with avidin conjugated with the Cy3 fluorochrome (Jackson ImmunoResearch Laboratories). Thereafter, the slides were dehydrated in a graded ethanol solutions (50°, 70° and 96°), dried, and mounted in DABCO (Merck) antifade solution containing 1 μg/ml DAPI*.*

### Histology

After dissection, the gonads were fixed in Bouin’s solution (Sigma), dehydrated in graded ethanol solutions (70–100%), immersed in xylene and mounted in paraffin. Tissue sections of 7 µm in thickness were deparaffinized in xylene, rehydrated and stained via a routine protocol according to the Mallory method^77^. Imaging was performed with an Axioskop microscope (Zeiss) using AxioVision Rel 4.8 software (Zeiss).

### Whole-mount fluorescence *in situ* hybridization

Prior to whole mount FISH, tissues were incubated in a 0.5% solution of Triton X100 in 1× PBS for 4–5 hours at RT, washed in 1× PBS for 15 min followed by impregnation with 50% formamide, 10% dextran sulphate, and 2× SSC (saline-sodium citrate buffer; 20 × SSC – 3 M NaCl 300 mМ Na_3_C_6_H_5_O_7_) for 3–4 hours at 37 °C. The hybridization mixture for the PCR-labelled probe contained 50% formamide, 2× SSC, 10% dextran sulphate, 20 ng/µl probe and a 10- to 50-fold excess salmon sperm DNA. For the oligonucleotide probe, the hybridization mixture contained 40% formamide, 2× SSC and 10% dextran sulphate, 20 ng/µl probe and a 10 to 50-fold excess of tRNA. Gonadal tissues were denatured at 82 °C for 15 min and incubated for 24 hours at RT. In the case of the directly labelled oligonucleotide probe, the tissues were washed in three changes of 2× SSC at 42 °C for 15 min each, followed by staining with DAPI (1 µg/µl) (Sigma) prepared in 1× PBS. In the case of the PCR-labelled probe, the tissues were washed in three changes of 0.2× SSC at 42 °C for 15 minutes each and blocked in 4× SSC containing 1% blocking reagent (Roche) in 4× SSC for 1 hour at RT. Biotin was detected by incubation with avidin conjugated with Cy3 or Alexa 488 (Jackson ImmunoResearch Laboratories) for 12 hours. The tissues were stained with DAPI (1 µg/µl) (Sigma) prepared in 1× PBS at RT overnight.

### Whole-mount immunofluorescence staining

Kinetochore proteins were detected by human CREST serum (Antibodies Incorporated), and Vasa was detected with the rabbit polyclonal DDX4 antibody [C1C3] (GeneTex) via 3D immunofluorescence staining. Prior to the addition of the antibodies, the tissues were incubated in a 0.5% solution of Triton X100 in 1× PBS for 4–5 hours at RT, washed in 1× PBS at RT and incubated for 1–2 hours in a 1% blocking solution (Roche) in 1× PBS. Incubation with the primary antibodies (concentration 1:40) was carried out at RT overnight, followed by washing in 1× PBS with 0.01% Tween (ICN Biomedical Inc). Secondary antibodies conjugated with the Alexa-488 fluorochrome were applied for 12 hours at RT. The tissues were then washed in 1× PBS with 0.01% Tween (ICN Biomedical Inc) and stained with DAPI (1 µg/µl) (Sigma) in 1× PBS at RT overnight.

### Immunofluorescence in tissue sections

Beta tubulin staining was carried out using rabbit polyclonal antibodies (ab6046, Abcam) following the protocol described in Chmielewska *et al*.^25^. Briefly, 20 µm-thick cryostat sections were mounted on Superfrost Plus microscope slides (Thermo Scientific), thawed and permeabilized for 1 hr and 15 min at RT in 0.5% Triton X-100 (Sigma) in 1× PBS after washing in PBST (pH 7.4, 14 mM NaCl, 0.27 mM KCl, 1 mM Na_2_HPO_4_, 0.18 mM KH_2_PO_4_, 0.05% Tween 20). Next, the tissues were blocked in 6% bovine serum albumin (BSA) diluted in PBS for 30 min at RT. Then, primary antibodies diluted 1:200 in 3% BSA in PBST were applied for 2 days at 4 °C, followed by washing in PBST with gentle shaking. Donkey anti-Rabbit Rhodamine Red-X secondary antibodies (1:100, Jackson ImmunoResearch) diluted in 3% BSA in PBST containing 0.5 µg/ml DAPI were applied overnight at RT, followed by washes in PBST. Sections were mounted in Vectashield antifade mounting medium (Vector Laboratories, Thermo Fisher Scientific).

### Confocal laser scanning microscopy

Tissues were placed in a drop of DABCO antifade solution containing 1 mg/ml DAPI, and confocal laser scanning microscopy was carried out using a Leica TCS SP5 microscope based on the Leica DMI 6000 CS inverted microscope (Leica Microsystems, Germany). Specimens were analysed using an HC PL APO 40× objective. Diode, argon and helium-neon lasers were used to excite the DAPI fluorescent dye and the Alexa488 and Cy3 fluorochromes, respectively. Images were captured using LAS AF software (Leica Microsystems, Germany). Confocal imaging of the tissue sections was performed using an Olympus FV1000 confocal microscope equipped with a UPLSAPO 60× oil lens. The 3D-volume rendering and surface reconstruction of confocal image stacks were performed in Imaris 7.7.1 (Bitplane). The image stacks used for reconstruction were cropped to the region of interest (ROI), preserving the image voxel dimensions set during image acquisition. Isosurfaces of multichannel images were created for each channel separately, applying automated thresholding parameters for channel intensity cutoffs. ROI isosurfaces were split into separate surface objects corresponding to individual nuclei (DAPI channel) or FISH signals (Cy3 channel). In some cases when two adjacent nuclei were inseparable as individual surface objects, isosurface reconstruction was performed via a “manual creation” tab. For the highlighted visualization of germ cells, only surface objects belonging to individual germ cells were retained in the reconstruction.

### Ethic Statement

Collected species are not listed by the IUCN Red list or CITES. Collection of adult frogs was approved by the Polish General and Regional Directorates for Environmental Protection (DOP-oz.6401.02.2.2013.JRO, DOP-oz.6401.02.2.2013.2014.JRO.as, WPN.6205.28.2014.IW.2, DZP-WG.6401.02.5.2015.JRO, WPN.6401.177.2016.IL). Techniques used in the capture, breeding, tissue sampling and euthanasia sought to minimize animal suffering and were in accordance with recommendations of the Herpetological Animal Care and Use Committee (HACC) of the American Society of Ichthyologists and Herpetologists. Each individual was anaesthetized by submersion in a 0,5% solution of 3-aminobenzoic acid ethyl ester (MS 222). All experimental procedures were accepted by the Local Commission for Ethics in Experiments on Animals in Wrocław, Poland (7/2013, 27/2016).

## Supplementary information

Supplementary Information.

## Data Availability

The authors state that all data necessary for confirming the conclusions presented in the article are represented fully within the article.
