# Author Correction: Micronuclei in germ cells of hybrid frogs from *Pelophylax esculentus* complex contain gradually eliminated chromosomes

**DOI:** 10.1038/s41598-020-76320-x

**Published:** 2020-11-11

**Authors:** D. Dedukh, S. Riumin, M. Chmielewska, B. Rozenblut-Kościsty, K. Kolenda, M. Kaźmierczak, A. Dudzik, M. Ogielska, A. Krasikova

**Affiliations:** 1 Saint-Petersburg State University, Saint-Petersburg, Russia; 2 Amphibian Biology Group, Department of Evolutionary Biology and Conservation of Vertebrates, Faculty of Biological Sciences, University of Wrocław, Wrocław, Poland

Correction to: *Scientific Reports* 10.1038/s41598-020-64977-3, published online 26 May 2020

The original version of this Article contained a typographical error in the spelling of the author M. Kaźmierczak, which was incorrectly given as M. Kazmierczak. This has now been corrected in the PDF and HTML versions of the Article, and in the accompanying Supplementary Information file.

