# Supplementary material for: Micronuclei in germ cells of hybrid frogs from Pelophylax esculentus complex contain gradually eliminated chromosomes
Source: Sci Rep. 2020 May 26;10:8720. doi: 10.1038/s41598-020-64977-3 (PMC7251083; doi:10.1038/s41598-020-64977-3)
Supplement: Supplementary file 1 — Supplementary Information. [file 41598_2020_64977_MOESM1_ESM.pdf]

Supplementary Materials

Micronuclei in germ cells of hybrid frogs from *Pelophylax esculentus* complex contain gradually eliminated chromosomes

Dedukh D, Riumin S, Chmielewska M, Rozenblut-Kościsty B, Kolenda K, Kazmierczak M, Dudzik A, Ogielska M, Krasikova A

Supplementary Figure 1. Gonocytes in the gonads of *P. ridibundus* (a) and diploid *P. esculentus* hybrids identified using anti-Vasa antibodies. Arrows indicate gonocytes. Arrowheads indicate micronuclei in the cytoplasm of gonocytes. Scale bars = 20 μm.

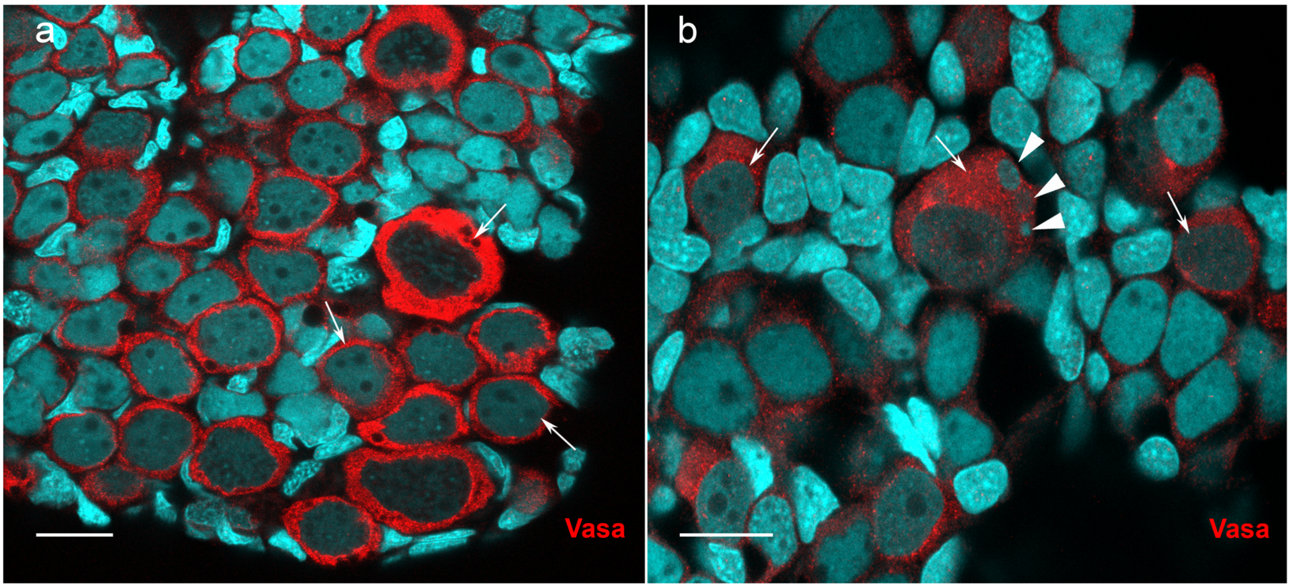
a
Vasa
b
Vasa

Supplementary Figure 2. Identification of the centromeric regions of all *P. ridibundus* chromosomes by FISH with a probe to centromeric RrS1 repeat. Metaphase chromosome spreads from *P. ridibundus* (a) and *P. esculentus* (b) are shown. Arrows indicate centromeric regions of *P. ridibundus* chromosomes. Scale bars = 10 μm.

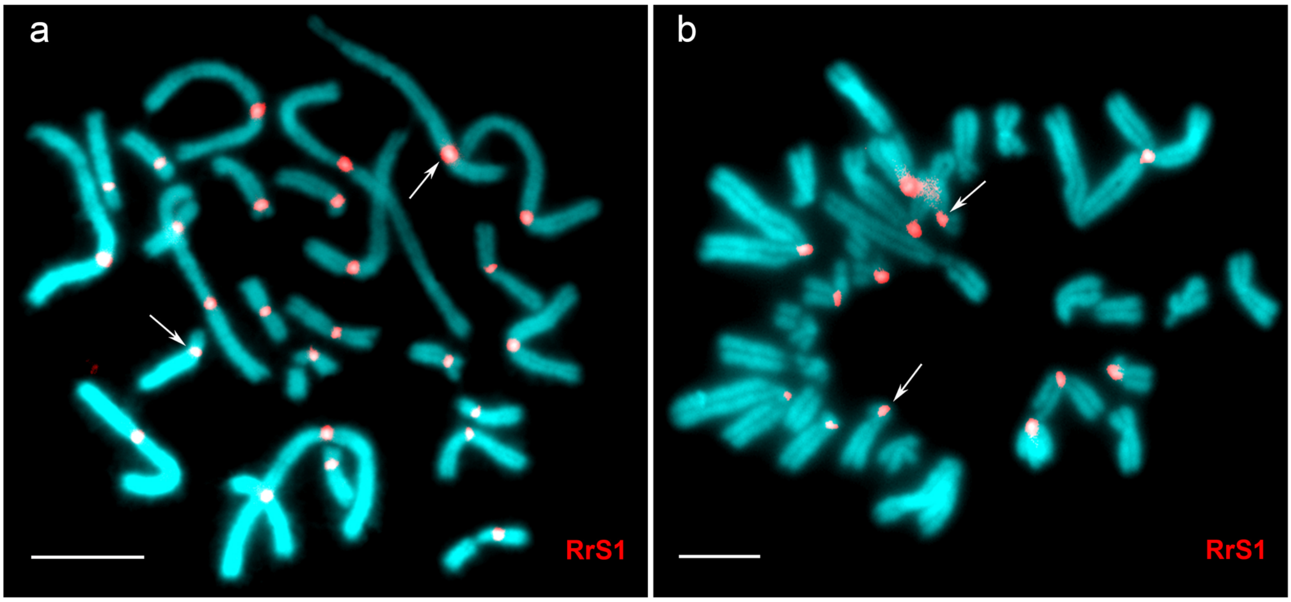
a
RrS1
b
RrS1

Supplementary Table 1. Tadpoles of water frogs from the *Pelophylax esculentus* complex used in the study.

Supplementary Table 1

3D-FISH RrS1-probe

Ploidy	Tadpoles IDs	Stage of tadpoles development according to Gosner (1960)	Genome composition	N of germ cells	N of germ cells with micronuclei	N of micronuclei	N of micronuclei RrS1(+)	Relative number of micronuclei with signals	Misaligned chromosomes	Signal in misaligned chromosomes	Interphase cells with 26 signals
3n	1.2/2016	29	LLR	329	70	77	4	5,2			
3n	1.3/2016	27-28	LLR	453	74	83	79	95,2			
3n	1.4/2016	26	LLR	179	64	93	90	96,8			
3n	1.6/2016	35	LLR	435	145	183	174	95,1			
3n	1.7/2016	34	LLR	683	149	205	191	93,2			
3n	1.8/2016	32	LLR	332	90	106	104	98,1			
3n	1.9/2016	30	LLR	261	59	91	88	96,7			
3n	1.10/2016	34	LLR	214	35	49	35	71,4	1 cell: 3 chromosomes	unclear	
3n	1.11/2016	31	LLR	689	103	140	101	72,1			
3n	1,13/2016	30-31	LLR	373	74	85	85	100,0			
3n	1.14/2016	29-30	LLR	481	96	134	125	93,3			
3n	1.17/2016	35	LLR	294	36	43	37	86,0			
3n	1.21/2016	33	LLR	61	8	9	5	55,6			
3n	1.25/2016	32-33	LLR	1395	167	224	139	62,1			
3n	1.x/2016		LLR	193	60	74	65	87,8			
3n	4.37/2016	39	LLR	663	88	122	101	82,8			
3n	4.11/2016	32-33	LLR	748	134	174	170	97,7			
3n	4.6/2016	30	LLR	654	101	148	139	93,9			
3n	4.13/2016	31	LLR	476	82	99	91	91,9			
3n	4.4/2016	32	LLR	285	99	145	140	96,6			
3n	4.19/2016	35	LLR	348	78	91	83	91,2			
2n	5.13/2016	32	RL	148	46	58	5	8,6			
2n	5.26/2016	33	RL	621	97	132	17	12,9			
2n	7.26/2016	26	RL	164	21	27	2	7,4			
2n	7.36/2016	30	RL	186	54	71	8	11,3			
2n	8.32/2016	28	RL	177	54	89	5	5,6			

2n	9.1/2016	33	RL	887	177	210	16	7,6	2 cells: 1 chromosome, 2 chromosome	no, 2	yes
2n	9.11/2016	28	RL	753	233	311	24	7,7			
2n	9.19/2016	27	RL	1278	356	421	25	5,9			
2n	9.25/2016	28	RL	1627	321	336	11	3,3			
2n	9.38/2016	29-30	RL	883	105	107	3	2,8			
2n	9.52/2016	33-34	RL	1378	279	315	18	5,7			
2n	9.57/2016	31	RL	521	145	173	11	6,4			
2n	9.60/2016	32	RL	1544	243	332	50	15,1	1 cell: 2 chromosomes	no	
2n	11.27/2016	28	RL	582	67	100	10	10,0			
2n	12.1/2016	36	RL	682	125	131	9	6,9			yes
2n	12.3/2016	35-36	RL	95	33	53	0	0,0			
2n	12.11/2016	36	RL	682	125	131	9	6,9			
2n	12.18/2016	25	RL	321	68	68	3	4,4			
2n	12.22/2016	26	RL	763	92	95	3	3,2			
2n	12.26/2016	26	RL	486	87	98	5	5,1			
2n	12.27/2016	26	RL	754	131	179	21	11,7			
2n	16.4/2016	32	RL	666	99	145	14	9,7			
2n	16.7/2016	33	RL	1402	245	300	33	11,0	2 cells: 1 chromosome, 1 chromosome	no, no	
2n	16.14/2016	29-30	RL	388	75	75	2	2,7			
2n	16.16/2016	32	RL	653	98	141	9	6,4			
2n	16.17/2016	33	RL	482	74	96	15	15,6			
2n	16.29/2016	33	RL	169	27	34	5	14,7	1 cell: 1 chromosome	no	yes
2n	16.30/2016	32	RL	993	172	241	54	22,4	1 cell: 1 chromosome	no	yes
2n	16.41/2016	33	RL	666	103	110	7	6,4			
2n	16.43/2016	33	RL	191	28	32	23	71,9			
2n	16.47/2016	32	RL	724	83	96	12	12,5			
2n	16.40/2016	31	RL	847	101	169	13	7,7			
2n	23.1/2016	33	RL	1328	431	763	28	3,7			
2n	23.2/2016	32	RL	956	114	197	17	8,6			
2n	23.11/2016	33-34	RL	138	20	26	4	15,4	1 cell: 1 cromosome	no	yes
2n	23.46/2016	34	RL	180	28	34	2	5,9			yes
2n	23.65/2016	34	RL	31	6	6	1	16,7			

2n	26.5/2016	33-34	RL	563	82	108	16	14,8			
2n	26.7/2016	34	RL	894	88	135	6	4,4			
2n	27.7/2016	30	RL	472	88	95	1	1,1			
2n	27.13/2016	34	RL	745	89	96	5	5,2			
2n	27.18/2016	33	RL	663	76	103	7	6,8			
2n	28.7/2016	32-33	RL	753	87	128	12	9,4			
2n	28.23/2016	33-34	RL	583	97	105	15	14,3			
2n	30.17/2016	26	RL	385	59	85	16	18,8			
2n	30.33/2016	32	RL	428	101	275	15	5,5			
2n	30.35/2016	30	RL	194	89	89	0	0,0			
2n	30.40/2016	30	RL	248	85	126	12	9,5			
2n	30.41/2016	29	RL	299	71	94	7	7,4			
2n	30.47/2016	31	RL	1403	61	71	19	26,8	2 cells: 1 chromosome, 1 chromosome	no, no	
2n	30.48/2016	33	RL	376	37	62	42	67,7			
2n	30.56/2016	31	RL	2039	110	132	88	66,7			
2n	37.3/2016	35	RL	38	7	7	0	0,0			yes
2n	37.9/2016	30	RL	678	53	61	8	13,1	1 cell: 1 chromosome	no	
2n	7.11/2017	39	RL	432	85	100	13	13,0			
2n	10.7/2017	39	RL	88	31	59	2	3,4			
2n	10.27/2017	38	RL	582	67	100	10	10,0			
2n	15.4/2017	30	RL	354	77	79	5	6,3			
2n	16.5/2017	32	RL	574	121	143	15	10,5	3 cell: 1 chromosome, 2 chromosomes, 1 chromosome	no, no, yes	
2n	16.6/2017	29	RL	653	86	100	8	8,0	1 cell: chromosome	no	
2n	17.37/2017	37-38	RL	389	85	109	13	11,9			yes
2n	17.x/2017	36	RL	146	76	88	7	8,0			yes
2n	17.33/2017	37	RL	196	69	88	11	12,5			yes

IF with CREST serum											
Ploidy	Tadpoles Ids	Stage of tadpoles development according to Gosner (1960)	Genome composition	N of germ cells	N of germ cells with micronuclei	N of micronuclei	N of micronuclei with CREST(+)				
3n	4.15/2016	36	LLR	76	21	26	24				
3n	4.x/2016		LLR	150	39	53	41				
2n	8.24/2016	26-27	RL	90	33	51	47				
2n	8.14/2016	26-27	RL	195	76	102	96				
2n	9.8/2017	27	RL	346	129	143	140				
2n	9.44/2017	33	RL	653	301	351	345				
2n	9.31/2017	34	RL	284	108	143	141				
2n	9.54/2017	35	RL	89	41	64	64				
2n	10.13/2017	37	RL	543	107	135	135				
2n	10.4/2017	27	RL	289	69	84	80				
2n	10.10/2017	41	RL	127	58	66	66				
2n	15.17/2017	31	RL	119	86	99	95				
2n	17.7/2017	36	RL	198	99	121	117				
IF against Vasa											
Ploidy	Tadpoles Ids	Stage of tadpoles development according to Gosner (1960)	N of germ cells								
2n	29.1/2016	33	RR								
2n	29.2/2016	29	RR								
2n	29.3/2016	32	RR								
2n	29.4/2016	32	RR								
2n	9.51/2017	36-37	RL								
2n	15.31/2017	29	RL								

IF against beta tubulin											
Ploidy	Tadpoles Ids	Stage of tadpoles development according to Gosner (1960)	Genome composition	Misaligned chromosomes							
2n	1126/22/2015	38	RL	1 cell: 2 chromosomes							
Histology											
Ploidy	Tadpoles Ids	Stage of tadpoles development according to Gosner (1960)	Genome composition	Misaligned chromosomes							
2n	488/25/2014/M	44	RL	1 cell: 2 chromosomes							
2n	709/2/2015/F	34	RL	1 cell: chromosome							

Supplementary Table 2. Parental individuals of water frogs from the *Pelophylax esculentus* complex used in crossing experiments.

Supplementary table 2						
**Crosses IDs**	**Sex**	**Adult frogs IDs**	**Genotype**	**PCR Pel SAI1**	**Habitat**	**Analyzed tadpoles genotype**
1/2016	female	1/16	RL	RL	Wysoka Kamieńska	LLR
	male	4/16	LL	LL	Sanie	
4/2016	female	1/16	RL	RL	Wysoka Kamieńska	LLR
	male	6/16	LLR	RKL	Wysoka Kamieńska	
5/2016	female	2/16	RL	KL	Uciechów	RL
	male	7/16	LLR	0	Uciechów	
7/2016	female	2/16	RL	KL	Uciechów	RL
	male	8/16	RR	0	Ruda Milicka	
8/2016	female	3/16	RL	KL	Uciechów	RL
	male	8/16	RR	0	Ruda Milicka	
9/2016	female	10/16	LL	LL	Sanie	RL
	male	9/16	RL	KL	Uciechów	
11/2016	female	12/16	RL	KL	Uciechów	RL
	male	13/16	LL	0	Sanie	
12/2016	female	12/16	RL	KL	Uciechów	RL
	male	14/16	LLR/RRL?	KL	Uciechów	
16/2016	female	18/16	RL	0	Uciechów	RL
	male	19/16	LL	0	Sanie	
23/2016	female	27/16	LL	0	Sanie	RL
	male	28/16	RL	0	Wysoka Kamieńska	
26/2016	female	34/16	LLR	0	Wysoka Kamieńska	RL
	male	32/16	RL	0	Wysoka Kamieńska	
27/2016	female	35/16	LLR	RKL	Wysoka Kamieńska	RL
	male	33/16	RL	RKL	Wysoka Kamieńska	
28/2016	female	36/16	LL	LL	Sanie	RL
	male	37/16	RL	RKL	Wysoka Kamieńska	
30/2016	female	38/16	RR	RR	Ruda Milicka	RL

	male	40/16	LLR	RL	Wysoka Kamieńska	
37/2016	female	50/16	RL	0	Uciechów	RL
	male	52/16	LL	0	Sanie	
7/2017	female	2/17	RL	0	Uciechów	RL
	male	18/17	LL	0	surroundings of Wrocław	
10/2017	female	1/17	LLR	0	Uciechów	RL
	male	11/17	RL	0	Uciechów	
15/2017	female	1/17	LLR	0	Uciechów	RL
	male	20/17	RR	0	Ruda Milicka	
16/2017	female	21/17	LL	0	Urwitałt, Mazury	RL
	male	20/17	RR	0	Ruda Milicka	
17/2017	female	21/17	LL	0	Urwitałt, Mazury	RL
	male	11/17	RL	0	Uciechów	
10/2016	female	10/16	LL	LL	Sanie	LL
	male	11/16	LL	LL	Sanie	
29/2016	female	38/16	RR	RR	Ruda Milicka	RR
	male	39/16	RR	RK	Ruda Milicka	
**IF on tissue sections**						
2/2015	female	6/2n/15 Bacz	RR	RR	Wysoka Kamienska	RL
	male	14/15	LL	LL	surroundings of Wrocław	
**Histology**						
25/2014	female	19/14	LL	LL	Urwitałt, Mazury	
	male	23/14	RR	RR	Ruda Milicka	
2/2015	female	5/15	RR	RR	Ruda Milicka	
	male	1/15	LL	LL	surroundings of Wrocław	
